# Discovery and Comparative Profiling of microRNAs in Representative Monopodial Bamboo (*Phyllostachys edulis*) and Sympodial Bamboo (*Dendrocalamus latiflorus*)

**DOI:** 10.1371/journal.pone.0102375

**Published:** 2014-07-11

**Authors:** Hansheng Zhao, Lili Wang, Lili Dong, Huayu Sun, Zhimin Gao

**Affiliations:** State Forestry Administration Key Open Laboratory on the Science and Technology of Bamboo and Rattan, International Center for Bamboo and Rattan, Beijing, China; St. Georges University of London, United Kingdom

## Abstract

**Background:**

According to the growth pattern of bamboo, sympodial bamboo and monopodial bamboo are considered as two mainly kinds of bamboo. They have different phenotypes and different characteristics in developmental stage. Much attention had been paid on the study of bamboo cultivation, processing, physiology, biochemistry and molecular biology, which had made great progresses in the last decade, especially for the highlighted achievement of the bamboo genomics. However, there is no information available on concerning comparative profiling of miRNAs between sympodial bamboo and monopodial bamboo, which might play important roles in the regulation of bamboo development.

**Methodology/Principal Findings:**

We identified the profiles of small RNAs using leaf tissues from one sympodial bamboo *i.e.* moso bamboo (*Phyllostachys edulis*) and another monopodial bamboo *i.e.* ma bamboo (*Dendrocalamus latiflorus*). The result showed that there were 19,295,759 and 11,513,888 raw sequence reads, in which 92 and 69 conserved miRNAs, as well as 95 and 62 novel miRNAs were identified in moso bamboo and ma bamboo, respectively. The ratio of high conserved miRNA families in ma bamboo is more than that in moso bamboo. In addition, a total of 49 and 106 potential targets were predicted in moso bamboo and ma bamboo, respectively, in which several targets for novel miRNAs are transcription factors that play important roles in plant development. More importantly, annotation of differentially expressed target genes was performed based on the analysis of pathway and gene ontology terms enrichment.

**Conclusions/Significance:**

This study provides the first large-scale sight of discovery and comparative characterization of miRNAomes between two representative bamboos belonged to sympodial bamboo and monopodial bamboo, respectively. Although it will be necessary to validate the function of miRNAs through more experimental research in further, these results lay a foundation for unraveling the miRNA-mediated molecular processes in different kinds of bamboo.

## Introduction/Background

MicroRNAs (miRNAs) are endogenous, non-coding small RNAs of approximately 22 nucleotides that regulate the flow of genetic information by controlling the translation or stability of mRNAs. It has been estimated that miRNAs account for ∼1% of predicted genes in higher eukaryotic genomes, and that up to 10–30% of genes may be regulated by miRNAs, which exist widely in animals, plants and some viruses. Since the discovery of the first miRNA in *Caenorhabditis elegans*, a large number of miRNAs have been identified by experimental cloning or bioinformatics methods with the development of biotechnology. More importantly, with the increasing development of high-throughput technology, the integrated analysis based on miRNA sequences in the evolutionary process provides a significant number of evidences that miRNAs evolved independently in different strains and generally were conserved in the process of evolution [1], [2]. However, some data and studies associated function with evolution for bamboo miRNAs have been rare till now.

As a tribe of flowering and evergreen perennial monocots, classified in the subfamily Bambusoideae within the grass family Poaceae that includes rice, maize, wheat and other cereals [3], bamboo represents one of the fastest-growing plants and one of the most important non-timber forest resources in the world [4], [5]. More importantly, bamboo is of notable economic and environmental significance, due to being used for versatile raw products. About 2.5 billion people depend on bamboo economically, and the international trade in bamboo amounts to over 2.5 billion US dollars per year [6].

Bamboo has rhizomes and roots underground that lives exclusively in forest and grows into large woody culms. There are two mainly patterns of bamboo for their growth, namely as sympodial bamboo and monopodial bamboo. Monopodial bamboo grows fast, through their roots or rhizome, which can spread widely underground and send up new culms to break through the surface. Therefore, this kind was named as running bamboo vividly. Monopodial bamboo ranges in all shapes and sizes, from a modest 30 cm (*i.e.* pygmy bamboo) to a mighty 18–20 m in height (*i.e.* moso bamboo). As one of significant bamboo in monopodial bamboo, moso bamboo (*Phyllostachys edulis*) is the most important economic one with many advantages such as fast growing rate, high yield, extensive use, short crucial period formation and strong regeneration capacity.

Sympodial bamboo grows like a tussock, and their new clums emerged around the outside edge of clump, which was aptly named as clumping bamboo. Due to unique root and rhizome structures, sympodial bamboo produces a tight cluster of culms and spreads only a couple centimeters out from the base each season. They range in height from less than 20 cm to more than 30 m, depending on different species of bamboo. Distinguished from monopodial bamboo, sympodial bamboo have some special features, such as lower spreading rate and u-shaped rhizome making new culms next to original plant. As one of the most popular and valuable bamboo species in sympodial bamboo, ma bamboo (*Dendrocalamus latiflorus*) fascinated people because of its evergreen color and delicious shoot, which was widely cultivated in southern China.

To facilitate omics studies in bamboo and help unveil the miRNAs feature of bamboo, small-RNA libraries of leaf tissues from moso bamboo and ma bamboo, respectively, were constructed and sequenced. In an attempt to identify new conserved miRNAs and novel miRNAs in bamboo, a total of 7,450 known miRNAs from 70 plants species from miRBase served as a reference data set. Moreover, the potential target genes of novel miRNAs were predicted based on the high-throughput technology and bioinformatics analysis. The profiling of microRNAs from moso bamboo and ma bamboo were compared. Ultimately, these results will play the necessary role in understanding the regulation of miRNAs in the development of monopodial bamboo and sympodial bamboo.

## Materials and Methods

### Plant material and RNA isolation

Divided seedlings of moso bamboo and ma bamboo were potted in our laboratory under a regime of 16 h light and 8 h darkness at 25°C, with a light intensity of 200 µmol·m^−2^·s^−1^ and a relative humidity of 75%. The leaf tissues were used as materials for this study. We chose the third piece of new functional leaf (blade tissue only) from the top of the branch, which could be considered as a juvenile leaf. The leaves were collected and quickly frozen in liquid nitrogen. Total RNA was isolated from leaf tissues using the Trizol reagent (Invitrogen, Carlsbad, CA, and USA), according to the manufacturer's instructions strictly.

### Small RNA high-throughput sequencing

The small RNA library construction and Solexa sequencing were carried out at BGI-Shenzhen (Shenzhen, China) using the standard small RNA sample preparation protocol (Illumia) as described by Hafner *et al.* [7]. Briefly, small RNAs of 15–30 nt in length were first isolated from the total RNA through 15% TBE urea denaturing polyacrylamide gels, which were ligated to the small RNA-5′ adaptor, and then 3′ adaptor was ligated to the small RNA-5′ adaptor, followed by reverse transcription into cDNAs. These cDNAs were amplified by PCR and subjected to Solexa sequencing.

### Sequence data analysis

After removing low quality reads and trimming adapter sequences, initial reads based on Solexa sequencing were processed by summarizing data production, evaluating sequencing quality, calculating the length distribution of small RNA reads. Moreover, small RNAs ranging from 18–30 nt were collected and used for further analyses. The raw reads are available in the NCBI SRA database under the accession number SRX480448. For analyzing conserved and novel miRNA, firstly, the clean reads, which mapped to exons or other non-coding RNAs such as rRNA, snRNA, snoRNA and tRNA, are filtered by BLAST against the coding sequences of moso bamboo genome, the Rfam database (http://rfam.sanger.ac.uk/) [8] and the GenBank non-coding RNA database (http://www.ncbi.nlm.nih.gov/) to discard coding gene, non-coding RNA sequences. Subsequently, unique sRNAs were aligned with plant mature miRNAs in miRBase Release 20. After rigorous screening, sRNA sequences without more than three mismatched bases were selected by BLAST searching against miRBase. Then, the remaining reads were used to map the genome of moso bamboo. Sequences with a tolerance of two mismatches were retained for miRNA prediction. RNAfold (http://www.tbi.univie.ac.at/RNA/) [9] was used for secondary structure prediction (hairpin prediction) of individual mapped miRNAs, using the default folding conditions to identify conserved miRNAs in bamboo. Lastly, sequences without defined as the conserved miRNAs were termed as novel miRNAs.

RPKM (Reads Per Kilobase per Million) is a method of quantifying gene expression from RNA sequencing data by normalizing for total read length and the number of sequencing reads [10]. Based on the same principle, RPKM is suitable the analysis of miRNA as well. The equation for calculating RPKM is in the following.
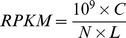



- C: the number of one reads mapped genome.

- N: the number of total reads mapped genome.

- L: the length of reads mapped genome.

In addition, to reasonably compare the miRNA abundance in libraries of moso bamboo and ma bamboo, the value of RPKM were calculated to seek the normalization of the difference between two samples. As a unique exact match, the information that a read mapped only once in the genome with no mismatch was also provided.

Potential target sequences for the novel miRNAs were predicted on the basis of the psRNATarget program (http://plantgrn.noble.org/psRNATarget/) with default parameters [11]. As the requirement of program, the newly identified miRNA sequences were used as custom miRNA sequences, while coding sequences in moso bamboo genome were used as custom plant databases. All predicted target genes were evaluated by the scoring system and criteria defined in a previous report [12].

To obtain custom enrichments, Gene Ontology (GO) terms enrichment was calculated by Ontologizer [13], which is a java application that can be used to perform statistical analysis for enrichment of GO terms in set of genes or proteins based on the one-sided Fisher's exact test, the novel parent-child method, and topology-based algorithms. As the inputs for Ontologizer, the four files were needed, including one GO file with the format of open biomedical ontologies, one list of all gene IDs of moso bamboo, one list of gene IDs that will be analyzed and one association file of moso bamboo. As parameter settings, term for term analysis with model-based gene set was used. GO terms analysis was performed separately on the set of target genes in sample of moso bamboo and ma bamboo. Besides, GO categories with p-values less than 0.05 were considered significant. Lastly, the biological interpretation of the target genes of differential miRNAs was completed using KEGG (Kyoto Encyclopedia of Genes and Genomes) pathway analysis.

### Expression analysis of miRNAs by qRT-PCR

To detect the expression level of miRNA in bamboo, stem-loop qRT-PCR was employed in this study. Primers were designed specifically based on individual miRNA using previous method [14]. U6 snRNA was selected as internal control [15]. More detailed information of primers was supported in File S1.

On the basis of leaf samples in moso bamboo and ma bamboo, cDNAs were synthesized from total RNA with the miRNA-specific stem-loop RT primer according to reference [16]. Subsequently, qRT-PCR was carried out using a SYBR Green I Master Kit (Roche, Germany) on a QTOWER2.2 Real-Time PCR System (Analytik Jena). The final volume was 10 µl, containing 5.0 µl 2×SYBR Premix Ex Taq, 0.2 µl of each primer (10 µM), 0.8 µl of cDNA and 3.8 µl of nuclease-free water. All reactions were repeated three times and the amplification was conducted as follows: initial denaturation at 95°C for 10 min, followed by 50 cycles at 95°C for 10 s, and 62°C for 10 s. There was three biological experiments. Expression levels were normalized to that of the internal control, and the relative value was calculated using previous method [17].

### MiRNA ioslation by PCR and sequencing

To prove the existence of miRNAs in bamboo, stem-loop primers were also directly used to amplify the mature miRNA sequences using specific templates from moso bamboo and ma bamboo respectively. Moreover, the primers designed for precursors (File S2) were used to amplify with DNA templates of moso bamboo and ma bamboo respectively. All the amplified products were sequenced and analyzed.

## Results and Discussion

### Summary of small RNA library sequencing

**Figure 1:**
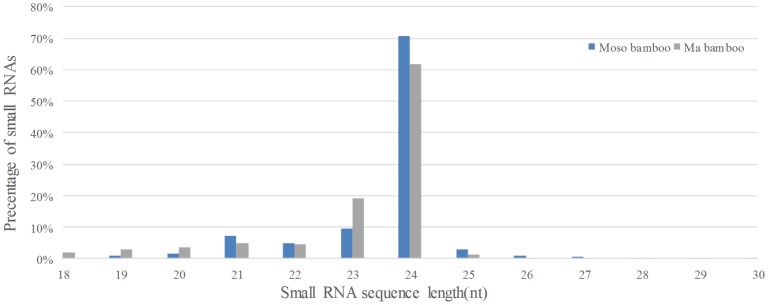
Length distribution of bamboo small RNA.

Based on Solexa sequencing of small RNA libraries from moso bamboo and ma bamboo (File S3), 19,295,759 and 11,513,888 raw reads were produced, respectively. After discarding low quality sequences and collapsed raw data, there were 8,089,795 (moso bamboo) and 10,593,305 (ma bamboo) clean reads as well as 2,653,717 (moso bamboo) and 6,320,379 (ma bamboo) unique sequences. Subsequently, the sequences, which mapped to coding sequences of moso bamboo genome and non-coding RNAs such as tRNAs, rRNAs, siRNAs, snRNA, and snoRNA, were removed. The statistics of small RNA library sequencing were shown in Table 1. As an important feature of the size profile, the distribution of small RNA length was summarized in Figure 1. The general distribution profile of moso bamboo was similar to that of ma bamboo. The most of small RNAs were ranged from 20 nt to 24 nt in length, which were highly consistent with those of small RNAs for known function [18]. As a maximum peak, small RNA with 24 nt in length was also highly agreed with those small RNAs in other Poaceae plants based on Solexa sequencing technology [19]–[21].

**Table 1:** Summary of small RNAs sequences from moso bamboo and ma bamboo.

Category	Unique sRNA				Total sRNA			
	Moso bamboo	Precent(%)	Ma bamboo	Precent(%)	Moso bamboo	Precent(%)	Ma bamboo	Precent(%)
**Protein coding gene (sense and antisense)**	227,278	8.56%	365,821	5.79%	1,123,397	13.89%	730,686	6.90%
only sense	125,845	4.74%	77,109	1.22%	324,341	4.01%	139,494	1.32%
only antisense	78,696	2.97%	211,602	3.35%	732,446	9.05%	451,698	4.26%
overlap (sense and antisense)	22,737	0.86%	77,110	1.22%	66,610	0.82%	139,494	1.32%
**Non-protein coding RNAs**	2,426,439	91.44%	5,954,558	94.21%	6,966,398	86.11%	9,862,619	93.10%
snRNA/snoRNA	5,240/2,870	0.20%/0.11%	11,820/6,852	0.19%/0.11%	11,291/6,744	0.14%/0.08%	16,124/9,255	0.15%/0.09%
sRNA	545	0.02%	1,079	0.02%	1,143	0.01%	1,716	0.02%
tRNA	525	0.02%	1,275	0.02%	1,738	0.02%	1,830	0.02%
rRNA	3,592	0.14%	7,591	0.12%	10,916	0.13%	9,971	0.09%
miRNA	2,138	0.08%	4726	0.07%	372,172	4.60%	85,698	0.81%
other sRNA	2,414,399	90.98%	5,928,067	93.79%	6,569,138	81.20%	9,747,280	92.01%
**Total**	2,653,717	100.00%	6,320,379	100.00%	8,089,795	100.00%	10,593,305	100.00%

### Identification of conserved miRNAs

We identified 92 and 69 conserved miRNAs, belonged to 53 families and 35 families for moso bamboo and ma bamboo, respectively, based on sequence alignment by BLAST against the latest miRBase v20, in which miRNAs of bamboo were no available, and a series of strict filtering criteria (File S4).

Normalized analysis of miRNA sequence between moso bamboo and ma bamboo were employed. The number of mapped reads, the length of mapped reads and the number of total mapped reads were considered to make it possible to compare RNA expression levels from different samples. In addition, the previously reports shown miRNAs with high sequencing frequencies played some key regulatory functions in maintaining biological process [22]. Consequently, the read counts for known miRNA families were evaluated in two samples. The result indicated small RNA families of moso bamboo were mapped to numerous conserved miRNA families, while those of ma bamboo were only concentrated in some conserved miRNA families. The reads with RPKM>5000 in two samples were focused on the abundance of conserved miRNA families, which accounted for a high proportion (∼99%) of the total conserved miRNA both in ma bamboo and moso bamboo. Apart from high abundant miRNA families, one must bear in mind that, there are a large number of low abundant miRNA families, indicating that these miRNAs were expressed at a low level. Another feature is that some conserved miRNAs were identified only in one sample. For example, miR396, miR397, miR1432 and miR7748 were identified only in moso bamboo, wherever miR170 was only in ma bamboo. This may be explained by the fact that the two kinds of bamboo had their own diversely biological and developmental features in the long progress of evolution. However, the previously study on the miRNA of ma bamboo demonstrated that miR396 family was identified [12]. It was may be caused by tissue and process specific miRNA in two different samples of ma bamboo [23], [24]. Taken together, as specific conserved miRNAs in moso bamboo, miR397, miR1432 and miR7748 were focused on using experimental validations further.

The number of members in different conserved miRNA families was also analyzed. The number of conserved miRNA families with more than 3 members was seven both in moso bamboo and ma bamboo. In addition, those with 3 members or less in both of moso bamboo and ma bamboo were accounted more than half of the total miRNA families.

### Evolution of miRNA in bamboo

**Figure 2:**
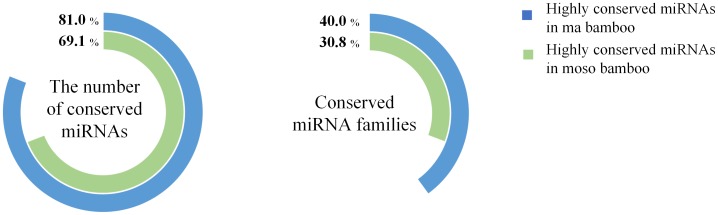
The ratio of high conserved miRNA between moso bamboo and ma bamboo.

Due to the important role of miRNA in regulation of plant gene expression, researchers have focused on the prediction, identification and functional analysis of miRNA in bamboo. However, the evolution of miRNA in bamboo has not been reported. To investigate the evolutionary roles of the conserved miRNAs, comparisons against conserved miRNAs in plants described by Cuperus *et al* [25] were carried out. The outcome showed almost of highly conserved miRNA families of bamboo were found (File S5), which further proved the previous arguments that miRNAs were highly conserved in sequences [26]. In addition, the previous study indicated that miRNA families from miR156 to miR408 were identified as high conserved miRNAs in the plant kingdom [27], [28]. These miRNAs play essential and conserved functions in plant development, such as flower and leaf development. MiRNAs families behind miR408 are low conserved or non-conserved, which may play roles in more species-specific characteristics in plant growth and development. These miRNA families were considered as the new generation in evolutional process. As shown in Figure 2, comparing conservation for miRNA families between two samples, a total of RPKM ranged from miR156 to miR408 accounted for 69.1%, 81.0% in moso bamboo and ma bamboo, respectively. Moreover, there is higher ratio of low conserved miRNAs families in moso bamboo (31%) than that of ma bamboo (19%). It was indicated that the implementation in regulatory processes, which played more essential roles, might need more involvement of miRNA in ma bamboo leaf than those in moso bamboo.

**Figure 3:**
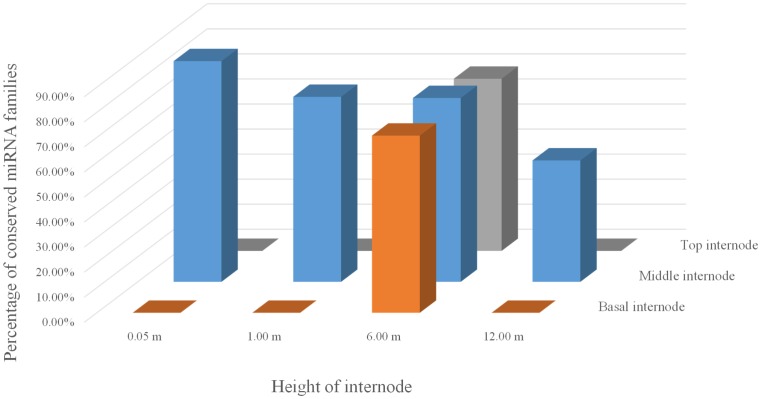
Proportion of conserved miRNAs in different unearthed shoots or different portions of moso bamboo.

The previously study demonstrated that miRNA sequences have to complement with their target gene transcripts to carry out their functions, which suggested that miRNA genes have to co-evolve with their target genes [29]. MiRNA genes would amplify through duplication events similar to those that drive the evolution of protein-coding genes: tandem gene duplications, segmental duplications, and chromosomal duplications or polyploidization [30], [31]. In this study, the different of proportion in low conserved miRNAs figures out more miRNAs with low conserved will be need to regulate newly emerging genes, which help moso bamboo adapt to the changing environments better than ma bamboo does [32], [33]. The rhizome of monopodial bamboo can spread laterally grown in soil, and also be differentiated into bamboo away from the mother plant, while sympodial bamboo grows in clusters within a relatively small range. Therefore, monopodial bamboo requires more self-regulation to adapt to the environment than sympodial bamboo, which might be consistent with the generation of lower conserved miRNAs families. Studies have shown that the expression of miRNAs is closely related with the developmental stages of plant. Based on the analysis of the previous study on the miRNAs of moso bamboo [34], the analysis based on percentages of conserved miRNA families in different development of internode demonstrated that highly conserved miRNAs may play more essential roles in earlier development of moso bamboo due to some stress response, while non-conserved miRNAs with low expression occupied a dominant position in maintaining the regulatory function during mature stage of moso bamboo (Figure 3). Moreover, the results based on percentages of conserved miRNA families in different positions of same development stage illustrated that highly conserved miRNAs from different positions of same tissue were possibly stable in one stage. To further understand the feature of bamboo, the related studies on other species of bamboo will be properly carried out.

### Identification of novel miRNAs

According to the analysis, we predicted 95 and 62 candidates of novel miRNAs with more than 3 reads in moso bamboo and ma bamboo, respectively. The mature sequences of novel miRNA with RPKM>300 in two samples were shown in Table 2. The comprehensive information on novel mature miRNAs was provided in File S6.

**Table 2:** Novel mature miRNAs with RPKM>300 in moso bamboo and ma bamboo.

Name	Novel mature miRNA					Precursor microRNA Position			
	Sequence	Length (nt)	Read count	RPKM	Unique mapped reads	Scaffold	Start	End	Strand
*phe*-miRC1-1	TTCAGGAGAGATGACACCGACA	22	16421	106143.04	Yes	PH01008094	15277	15526	+
*phe*-miRC1-2	TTCAGGAGAGATGACACCGACA	22	16421	106143.04	Yes	PH01008094	15158	15407	+
*phe*-miRC2-1	TTCAGGAGAGATGACACCGAC	21	6798	46033.761	Yes	PH01008094	15277	15526	+
*phe*-miRC2-2	TTCAGGAGAGATGACACCGAC	21	6798	46033.761	Yes	PH01008094	15158	15407	+
*phe*-miRC3-1	TTCAGGAGAGATGACACCGACAT	23	623	3851.8982	Yes	PH01008094	15277	15526	+
*phe*-miRC3-2	TTCAGGAGAGATGACACCGACAT	23	623	3851.8982	Yes	PH01008094	15158	15407	+
*phe*-miRC4	TGGACGTGAAGAGTGCTTTCC	21	368	2491.9718	No	PH01058200	243	492	+
*phe*-miRC5-1	AGAAGAGAGAGAGTACAGCTT	21	172	1164.7259	Yes	PH01000671	515204	515453	−
*phe*-miRC5-2	AGAAGAGAGAGAGTACAGCTT	21	172	1164.7259	Yes	PH01008818	10132	10381	+
*phe*-miRC5-3	AGAAGAGAGAGAGTACAGCTT	21	172	1164.7259	Yes	PH01257637	66	315	+
*phe*-miRC6-1	TTCAGGAGAGATGACACCGA	20	123	874.5602	Yes	PH01008094	15277	15526	+
*phe*-miRC6-2	TTCAGGAGAGATGACACCGA	20	123	874.5602	Yes	PH01008094	15158	15407	+
*phe*-miRC7	AAGCAATTAGACTGTTCTGGC	21	110	744.88286	No	PH01224661	328	577	+
*phe*-miRC8	TCGTCGTACGATTTAAGTGGTA	22	90	581.74736	No	PH01156600	211	460	−
*phe*-miRC9	AAGCAATTAGACTGTTCTGGCA	22	63	407.22315	No	PH01224661	328	577	+
*phe*-miRC10-1	CAAGCAATTAGACTGTTCTGGCA	23	55	340.05522	No	PH01224661	328	577	+
*phe*-miRC10-2	TGTGTTCAGGAGAGATGACAC	21	50	338.58312	Yes	PH01008094	15277	15526	+
*phe*-miRC10-3	TGTGTTCAGGAGAGATGACAC	21	50	338.58312	Yes	PH01008094	15158	15407	+
*phe*-miRC11-1	CACAAAGCAAATGGTACACTCTAG	24	57	337.73666	Yes	PH01002408	184744	184993	+
*phe*-miRC11-2	CACAAAGCAAATGGTACACTCTAG	24	57	337.73666	Yes	PH01005297	4205	4454	+
*phe*-miRC11-3	CACAAAGCAAATGGTACACTCTAG	24	57	337.73666	Yes	PH01037915	669	918	+
*phe*-miRC11-4	CACAAAGCAAATGGTACACTCTAG	24	57	337.73666	Yes	PH01059453	575	824	+
*phe*-miRC11-5	CACAAAGCAAATGGTACACTCTAG	24	57	337.73666	Yes	PH01059453	1131	1380	+
*phe*-miRC11-6	CACAAAGCAAATGGTACACTCTAG	24	57	337.73666	Yes	PH01079235	46	295	+
*phe*-miRC12-1	AACGAACTGACGAGTTGAGGGACG	24	53	314.03584	Yes	PH01080001	568	817	−
*phe*-miRC12-2	AACGAACTGACGAGTTGAGGGACG	24	53	314.03584	Yes	PH01080001	487	736	−
*phe*-miRC13-1	TTCAGGAGAGATGACACCGACATC	24	51	302.18543	Yes	PH01008094	15277	15526	+
*phe*-miRC13-2	TTCAGGAGAGATGACACCGACATC	24	51	302.18543	Yes	PH01008094	15158	15407	+
*dla*-miRC1-1	AGAAGAGAGAGAGTACAGCTT	21	101	3020.644	No	PH01000671	515204	515453	−
*dla*-miRC1-2	AGAAGAGAGAGAGTACAGCTT	21	101	3020.644	No	PH01008818	10132	10381	+
*dla*-miRC1-3	AGAAGAGAGAGAGTACAGCTT	21	101	3020.644	No	PH01257637	66	315	+
*dla*-miRC2	CTCAACCGTTGGGCGACTGCA	21	72	2153.3304	Yes	PH01004844	10351	10600	−
*dla*-miRC3	CTGTTGGTGTAGGGACAGATGCAT	24	40	1046.7578	Yes	PH01003884	56289	56538	+
*dla*-miRC4	TGCAGTGGACTGTGCAACACCGGA	24	37	968.251	Yes	PH01174398	1	236	+
*dla*-miRC5	CCTAATGGTTGGGCGACTGGAGGC	24	26	680.39259	Yes	PH01005539	882	1131	−
*dla*-miRC6	AGGTGCAGTGGCAGATGCAGC	21	17	508.42523	Yes	PH01006601	674	923	−
*dla*-miRC7-1	GAAGAGAGAGAGTACAGCTT	20	16	502.44376	No	PH01000671	515204	515453	−
*dla*-miRC7-2	GAAGAGAGAGAGTACAGCTT	20	16	502.44376	No	PH01008818	10132	10381	+
*dla*-miRC7-3	GAAGAGAGAGAGTACAGCTT	20	16	502.44376	No	PH01257637	66	315	+
*dla*-miRC8	GATATGCGGTTGTAGTAGATTGGA	24	19	497.20997	No	PH01004765	60417	60666	−
*dla*-miRC9	TGCAGTGGACTGTGCAACACCGG	23	18	491.52107	Yes	PH01174398	1	236	+
*dla*-miRC10	AAGTGCATGGTAGAGCGGTAGACC	24	17	444.87208	Yes	PH01003867	107894	108143	−
*dla*-miRC11-1	CGCTGTACGGTTGGGCGACTGCAA	24	16	418.70313	Yes	PH01144019	610	859	−
*dla*-miRC11-2	CGCTGTACGGTTGGGCGACTGCAA	24	16	418.70313	Yes	PH01144019	428	677	−
*dla*-miRC12	GCCTAATGGTTGGGCGACTGGAGG	24	15	392.53419	Yes	PH01005539	882	1131	−
*dla*-miRC13	GCAGTGGACTGTGCAACACCGGA	23	14	382.29417	Yes	PH01174398	1	236	+
*dla*-miRC14	CCTAAGGCGTGGAATAGAAGTGGA	24	13	340.1963	Yes	PH01085250	495	744	−
*dla*-miRC15-1	AGACTTGAAGCCATTAGAATC	21	11	328.98103	No	PH01086530	708	957	−
*dla*-miRC15-2	TGTAATGTTGTAGAAGATGAGGAG	24	11	328.98103	Yes	PH01018590	1476	1725	−

The number of novel miRNAs and unique mapped reads of ma bamboo were less than those of moso bamboo, probably because the genome of moso bamboo was analyzed as required genome. Although both of moso bamboo and ma bamboo belonged to Bambusoideae, phylogenetic analyses between these had few study for the lack of the genome information of ma bamboo. Therefore, using the genome of moso bamboo for analyzing miRNA of ma bamboo would inevitably produce some biases. To address the gap, the whole genome sequencing of ma bamboo need be implemented as soon as possible.

Among the novel miRNAs, *phe*-miRC1-1 with 106143.04 RPKM, more than 3-folds of the first-highest in ma bamboo, had the highest expression level in moso bamboo. On the basis of their frequencies and sequences in the small RNA libraries, although the expression levels of these candidates ranged from thousands of RPKM to lower, in general, novel miRNA candidates showed lower expression level compared with most of the conserved families. The low abundance of novel miRNAs demonstrated that these miRNAs might play a specific and essential role in certain tissues or developmental stages, and were considered as young miRNAs in terms of evolution [25].

### Prediction of miRNA targets

The acquisition of knowledge for miRNA target genes contributed to insight into the range of miRNA regulation and unveiling detailed descriptions of miRNA-target interactions. In addition, as an essential step to identify miRNA targets, the methods based on bioinformatics were widely used [11]. Herein, on the basis of the criteria described in the material and methods, a total of 49 and 106 target genes were identified in moso bamboo and ma bamboo, respectively. The target genes of novel miRNAs with RPKM>300 in two samples were shown in Table 3. The comprehensive information on target genes was provided in File S7.

**Table 3:** The target genes of Novel miRNAs with RPKM>300 in moso bamboo and ma bamboo.

miRNA name	Target ID	Inhibition	Target annotation
*phe-miRC5-1*	PH01000594G0440	Cleavage	SPL14 - SBP-box gene family member
*phe-miRC5-1*	PH01000770G0270	Cleavage	SPL17 - SBP-box gene family member
*phe-miRC5-1*	PH01000150G0460	Cleavage	SPL14 - SBP-box gene family member
*phe-miRC5-1*	PH01000050G0170	Cleavage	SPL17 - SBP-box gene family member
*phe-miRC5-1*	PH01003178G0220	Cleavage	SPL2 - SBP-box gene family member
*phe-miRC5-1*	PH01000002G1660	Cleavage	SPL2 - SBP-box gene family member
*phe-miRC5-1*	PH01000969G0180	Cleavage	SPL16 - SBP-box gene family member
*phe-miRC5-1*	PH01002673G0070	Cleavage	SPL16 - SBP-box gene family member
*phe-miRC5-1*	PH01000117G1390	Cleavage	SPL16 - SBP-box gene family member
*phe-miRC5-1*	PH01003773G0220	Cleavage	SPL16 - SBP-box gene family member
*phe-miRC7*	PH01000316G0850	Cleavage	PPR repeat containing protein
*phe-miRC9*	PH01000316G0850	Cleavage	PPR repeat containing protein
*phe-miRC11-1*	PH01000022G2070	Cleavage	SAC3/GANP family protein
*phe-miRC10-1*	PH01000316G0850	Cleavage	PPR repeat containing protein
*dla-miRC1-1*	PH01000594G0440	Cleavage	OsSPL14 - SBP-box gene family member
*dla-miRC1-1*	PH01000770G0270	Cleavage	OsSPL17 - SBP-box gene family member
*dla-miRC1-1*	PH01000150G0460	Cleavage	OsSPL14 - SBP-box gene family member
*dla-miRC1-1*	PH01000050G0170	Cleavage	OsSPL17 - SBP-box gene family member
*dla-miRC1-1*	PH01003178G0220	Cleavage	OsSPL2 - SBP-box gene family member
*dla-miRC1-1*	PH01000002G1660	Cleavage	OsSPL2 - SBP-box gene family member
*dla-miRC1-1*	PH01000969G0180	Cleavage	OsSPL16 - SBP-box gene family member
*dla-miRC1-1*	PH01002673G0070	Cleavage	OsSPL16 - SBP-box gene family member
*dla-miRC1-1*	PH01000117G1390	Cleavage	OsSPL16 - SBP-box gene family member
*dla-miRC1-1*	PH01003773G0220	Cleavage	OsSPL16 - SBP-box gene family member
*dla-miRC2*	PH01002521G0110	Cleavage	acyl-desaturase, chloroplast precursor
*dla-miRC4*	PH01000371G0630	Cleavage	expressed_protein
*dla-miRC8*	PH01002105G0330	Cleavage	OsGrx_C3 - glutaredoxin subgroup I
*dla-miRC8*	PH01001437G0080	Cleavage	regulator of nonsense transcripts 1
*dla-miRC8*	PH01000553G0260	Cleavage	regulator of nonsense transcripts 1
*dla-miRC9*	PH01000371G0630	Cleavage	expressed_protein
*dla-miRC10*	PH01000061G0870	Cleavage	tRNA synthetase class I
*dla-miRC10*	PH01000087G1230	Cleavage	expressed_protein
*dla-miRC6*	PH01000421G0420	Cleavage	AMP deaminase
*dla-miRC6*	PH01002076G0320	Cleavage	expressed_protein
*dla-miRC6*	PH01003497G0060	Cleavage	heavy metal-associated domain containing protein
*dla-miRC6*	PH01004252G0100	Translation	wall-associated receptor kinase 3 precursor
*dla-miRC6*	PH01003898G0120	Translation	zinc finger, ZZ type family protein
*dla-miRC6*	PH01002588G0080	Cleavage	expressed_protein
*dla-miRC6*	PH01004366G0020	Cleavage	DDT domain containing protein
*dla-miRC6*	PH01000778G0060	Cleavage	DDT domain containing protein
*dla-miRC6*	PH01001900G0280	Cleavage	expressed_protein
*dla-miRC6*	PH01001125G0220	Cleavage	OsSCP19 - Putative Serine Carboxypeptidase homologue
*dla-miRC6*	PH01002299G0250	Cleavage	coatomer subunit delta
*dla-miRC7-1*	PH01000594G0440	Cleavage	OsSPL14 - SBP-box gene family member
*dla-miRC7-1*	PH01000770G0270	Cleavage	OsSPL17 - SBP-box gene family member
*dla-miRC7-1*	PH01000150G0460	Cleavage	OsSPL14 - SBP-box gene family member
*dla-miRC7-1*	PH01000050G0170	Cleavage	OsSPL17 - SBP-box gene family member
*dla-miRC7-1*	PH01003178G0220	Cleavage	OsSPL2 - SBP-box gene family member
*dla-miRC7-1*	PH01000002G1660	Cleavage	OsSPL2 - SBP-box gene family member
*dla-miRC7-1*	PH01134604G0010	Cleavage	transferase family protein
*dla-miRC7-1*	PH01000197G0200	Cleavage	sarcoma antigen NY-SAR-91
*dla-miRC7-1*	PH01001526G0360	Cleavage	sister chromatid cohesion 2
*dla-miRC13*	PH01000733G0330	Cleavage	HAD-superfamily hydrolase, subfamily IA, variant 3 containing protein
*dla-miRC13*	PH01000707G0040	Cleavage	oxidoreductase, short chain dehydrogenase/reductase family protein
*dla-miRC14*	PH01000031G1370	Cleavage	tetratricopeptide repeat domain containing protein

The target genes of the novel miRNAs from bamboo mainly concentrated on transcription factor, signal regulation and functional protein, among which were a considerable number of *SPL* genes, indicating that these miRNAs might be involved in regulating the expression of *SPL* genes. *SPL* genes family is a group of structurally diverse genes encoding putative transcription factors found in photosynthetic organisms [35], [36]. The distinguishing feature of the *SPL* gene family is the SBP-box encoding a conserved protein domain of 76 amino acids in length. Recent studies indicated that SBP-box genes played central roles in controlling certain homeostatic processes, as well as in overcoming stresses [37], [38]. In the previous study of Arabidopsis, several targeted *SPL* genes act redundantly in controlling the juvenile-to-adult phase transition, which renders plants sensitive to photoperiodic induction of flowering [39], [40]. Therefore, the miRNAs targeted *SPL* genes might play important roles in bamboo leaves, which response to environmental factors especially for light induction.

### Annotation of differentially expressed target genes

Functional annotations were carried out to investigate which processes are differentially regulated by miRNA in moso bamboo and ma bamboo. To infer significant associations between the sets of targets predicted by individual miRNAs and specific biochemical pathways, a total of 20 different metabolic pathways were found, including 5 metabolism, 8 cellular processes, 7 genetic information processing (Table 4), which indicated that miRNA played essential roles in various biological process.

**Table 4:** List of the KEGG pathways of miRNA target genes affiliated.

KEGG Pathway			No. of target genes	Target genes	miRNAs
Cellular Processes	Cell growth and death	Cell cycle	4	PH01001634G0210, PH01000421G0420, PH01003083G0110, PH01000303G0070	*phe-miRC20, dla-miRC6, dla-miRC34-1, dla-miRC34-1*
Cellular Processes	Cell growth and death	Oocyte meiosis	4	PH01001634G0210, PH01000421G0420, PH01003083G0110, PH01000303G0070	*phe-miRC20, dla-miRC6, dla-miRC34-1, dla-miRC34-1*
Cellular Processes	Transport and catabolism	Peroxisome	2	PH01002603G0080, PH01002603G0080	*dla-miRC22, dla-miRC35*
Environmental Information Processing	Signal transduction	TGF-beta signaling pathway	2	PH01003083G0110, PH01000303G0070	*dla-miRC34-1, dla-miRC34-1*
Environmental Information Processing	Signal transduction	Wnt signaling pathway	2	PH01003083G0110, PH01000303G0070	*dla-miRC34-1, dla-miRC34-1*
Genetic Information Processing	Folding, sorting and degradation	Protein processing in endoplasmic reticulum	4	PH01000063G1430, PH01002461G0110, PH01003083G0110, PH01000303G0070	*phe-miRC39, dla-miRC28, dla-miRC34-1, dla-miRC34-1*
Genetic Information Processing	Folding, sorting and degradation	Ubiquitin mediated proteolysis	3	PH01000421G0420, PH01003083G0110, PH01000303G0070	*dla-miRC6, dla-miRC34-1, dla-miRC34-1*
Genetic Information Processing	Translation	mRNA surveillance pathway	2	PH01000553G0260, PH01000553G0260	*dla-miRC8, dla-miRC37*
Genetic Information Processing	Translation	RNA transport	2	PH01000553G0260, PH01000553G0260	*dla-miRC8, dla-miRC37*
Metabolism	Amino acid metabolism	Cysteine and methionine metabolism	1	PH01001446G0280	*dla-miRC40*
Metabolism	Amino acid metabolism	Valine, leucine and isoleucine degradation	1	PH01001555G0230	*dla-miRC47*
Metabolism	Carbohydrate metabolism	Pyruvate metabolism	1	PH01002248G0250	*phe-miRC65*
Metabolism	Energy metabolism	Carbon fixation in photosynthetic organisms	1	PH01002248G0250	*phe-miRC65*
Metabolism	Metabolism of cofactors and vitamins	Ubiquinone and other terpenoid-quinone biosynthesis	2	PH01001360G0010, PH01001360G0010	*dla-miRC21, dla-miRC38*

Besides pathway analysis, another functional annotation was performed via the analysis of GO, which is the *de facto* standard in gene functionality description and is used widely in functional annotation and enrichment analysis [41]. Genes from a large amount of organisms have been annotated to GO terms. The widespread applications based on GO terms enrichment analysis were the identification of annotation-enriched GO terms in a list of genes with some similar characteristics in biology. These terms were often considered as some representations with the outstanding biological features of the genes in the study of set [13]. We performed GO analysis on the result of prediction of miRNA targets using model-based gene set analysis (MGSA), which analyzes all GO terms at once by embedding them in a Bayesian network in order to provide high-level, summarized views of core biological processes.

**Figure 4:**
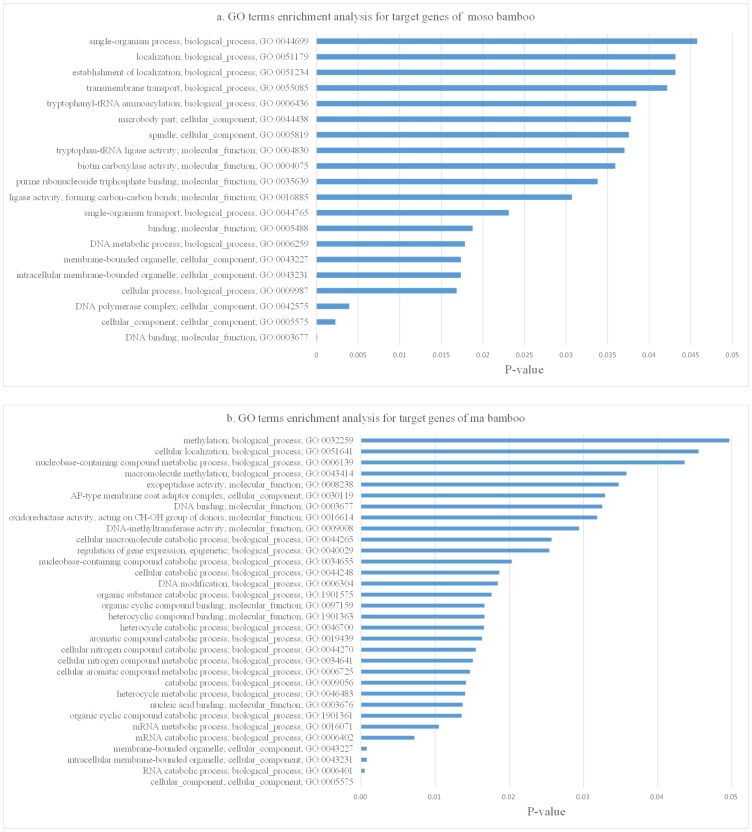
GO terms enrichment analysis for target genes between moso bamboo and ma bamboo.

In order to identify significant target genes, the GO term for target genes with p-value <0.05 were selected and shown in Figure 4. GO term enrichment analysis identified a total of 20 and 32 GO terms with high significant in moso bamboo and ma bamboo, respectively. The most highly ranked term of the target genes with higher significant in moso bamboo was DNA binding (GO: 0003677), which included 17 GO terms from the total 100 GO terms in the set of the moso bamboo. On the other hand, the most highly ranked term for the genes with significant in ma bamboo was cellular component (GO: 0005575) including 19 ones of the total 69 GO terms in the set of ma bamboo. Moreover, there were 4 GO terms overlapped (GO: 0003677, GO: 0005575, GO: 0043231 and GO: 0043227) in the total of significantly GO terms, of which 3 GO terms were distributed in cellular component part (GO: 0005575, GO: 0043231 and GO: 0043227). Furthermore, more highly ranked terms for the targets genes with higher expression were both in biological process, based on the significant analysis of GO terms between moso bamboo and ma bamboo. For example, on account of the category of GO terms, the ratio of biological process, cellular component and molecular function in moso bamboo was 40%, 30% and 30% of the total of GO terms, respectively, while the corresponded ratio were change to 65.6%, 12.5% and 21.9% in ma bamboo. Higher proportion of biological process in ma bamboo possibly reflected the significance of target genes, which might play more essential role than those in moso bamboo.

### Validation of specific conserved miRNAs by qRT-PCR and sequencing

**Figure 5:**
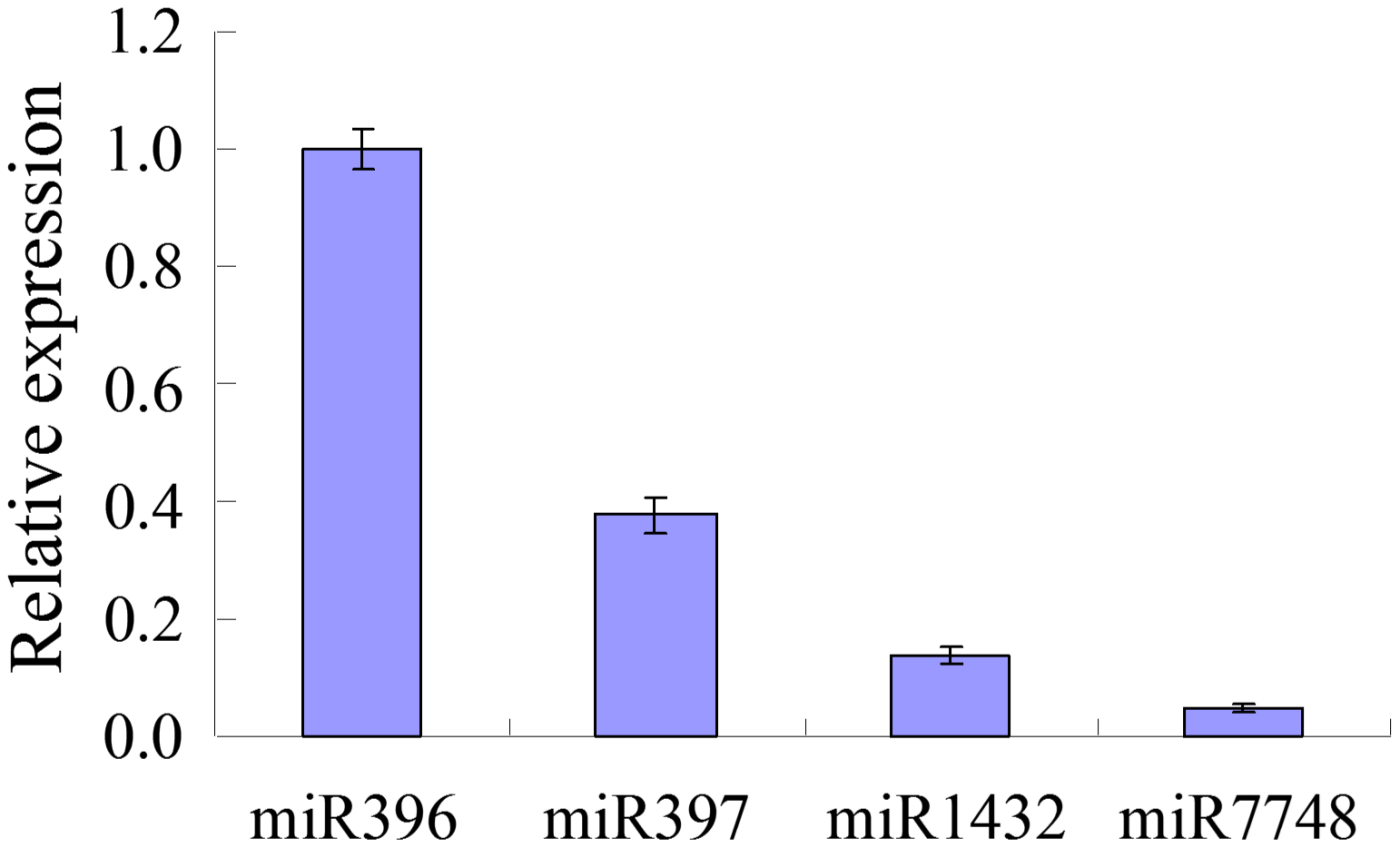
Expression analysis of miRNAs in leaves of moso bamboo using qRT-PCR. Error bars representing the standard deviation were derived from the three experiments in triplicate.

Specific primers designed for mature miRNA were used to validate the expression of miR396, miR397, miR1432, and miR7748 in leaves of both moso bamboo and ma bamboo. The result of qRT-PCR demonstrated that miR396, miR397, miR1432, and miR7748 were expressed differently in leaf of moso bamboo, among which miR396 was the highest one, followed by miR1432 and miR397, and miR7748 was the lowest one (Figure 5).

While that in ma bamboo, miR397 was the highest one, followed by miR1432 and miR396, miR7748 was not detectable (File S8). To prove the existence of miRNAs in ma bamboo, stem-loop primers were also directly used to amplify the mature miRNA sequences using specific templates from moso bamboo and ma bamboo, respectively, and the amplified products were sequenced. The result showed the sequences of mature miRNA in moso bamboo were all consistent with the sequences of miR396, miR397, miR1432, and miR7748 correspondingly. However, only three pairs of primers had specific amplification products for ma bamboo, one is the sequence of miR396,consistented with that of moso bamboo, while the other sequences were mismatched with miR397 and miR1432, respectively (File S9), which indicated the qRT-PCR result of miR397 and miR1432 for ma bamboo is false positive.

Moreover, the primers designed for precursors of miR396, miR397, miR1432, and miR7748 were used to amplify with DNA templates of moso bamboo and ma bamboo, respectively. The sequencing result demonstrated the sequences of moso bamboo were matched with the genomic sequences and could form the stem-loop structures (File S10). However, there were no specific PCR products from ma bamboo. In order to identify the targets of these miRNAs, we had screened the published ma bamboo data of transcriptome [42] and ESTs [43] in NCBI and bambooGDB database [44] using the targets of miR396, miR397, miR1432, and miR7748, respectively, and no matched sequences was found for them, which indicated that the target genes in moso bamboo and ma bamboo are not comparable. On the basis of these results, miR397, miR1432, and miR7748 were specific conserved in the leaf sample of moso bamboo.

## Conclusions

To the best of our knowledge, this work presents the firstly comparative profiling of microRNAs in representative monopodial bamboo (moso bamboo) and sympodial bamboo (ma bamboo), based on high-throughput sequencing analysis of small RNA. A large number of conserved miRNAs, novel miRNAs and target genes were identified, which are worthy of further investigation to improve our understanding of the regulatory mechanisms of miRNA in biological processes in bamboo. Target prediction of differential miRNAs indicated that *SPL* genes are mainly regulated by miRNAs. Functional annotation of genes targeted by differential miRNAs revealed that high significant biological processes are transcription regulation and photosynthesis. The expression of novel miRNAs and target genes involved in developmental stage of bamboo were further validated. Experiments showed that miR397, miR1432, and miR7748 were specific conserved in the leaf sample of moso bamboo. Taken together, the comparison between moso bamboo and ma bamboo indicated that monopodial bamboo and sympodial bamboo may be share some different miRNAs and theirs target genes in order to better adapt the development in different stages and stress response in their diverse course of evolution. Therefore, this study should lay an important foundation for future genetic or genomic studies on bamboo and help to fill the gap in comparative profiling of microRNAs.

## Supporting Information

File S1 **Primers for qRT-PCR and PCR of miRNAs.**
(DOC)

File S2 **Primers for the precursor amplification of miRNAs.**
(DOC)

File S3 **Dramatically different phenotype between moso bamboo and ma bamboo.**
(TIF)

File S4 **Conserved miRNAs in moso bamboo and ma bamboo.**
(XLSX)

File S5 **Heat map of highly conserved miRNA family in plants.**
(TIF)

File S6 **Novel mature miRNAs in moso bamboo and ma bamboo.**
(XLSX)

File S7 **miRNAs target genes in moso bamboo and ma bamboo.**
(XLSX)

File S8 **Expression analysis of miRNAs in leaves of ma bamboo using qRT-PCR.**
(DOC)

File S9 **Sequencing results of mature miRNAs.**
(DOC)

File S10 **Sequencing result of sequence containing miRNA precursor in moso bamboo.**
(DOC)

## References

[1] Floyd SK, Bowman JL (2004) Gene regulation: ancient microRNA target sequences in plants. Nature 428 (6982) 485–486. 15057819 10.1038/428485a

[2] Arteaga-Vazquez M, Caballero-Perez J, Vielle-Calzada JP (2006) A family of microRNAs present in plants and animals. Plant Cell 18 (12) 3355–3369. 17189346 10.1105/tpc.106.044420 PMC1785418

[3] Han JG, Xia DL, Li LB, Sun L, Yang K, et al (2009) Diversity of culturable bacteria isolated from root domains of moso bamboo (*Phyllostachys edulis*). Microbial ecology 58: 363–373. 19224269 10.1007/s00248-009-9491-2

[4] Zhou BZ, Fu MY, Xie JZ, Yang XS, Li ZC (2005) Ecological functions of bamboo forest: research and application. Journal of Forestry Research 16: 143–147.

[5] Peng ZH, Lu TT, Li LB, G ZM, Hu T, et al (2010) Genome-wide characterization of the biggest grass, bamboo, based on 10,608 putative full-length cDNA sequences. BMC Plant Biology 10: 116. 20565830 10.1186/1471-2229-10-116 PMC3017805

[6] Lobovikov M, Paudel S, Piazza M, Ren H, Wu JQ (2007) World Bamboo Resources: A thematic study prepared in the framework of the global forest resources assessment 2005. Non-wood Forest Products, Vol. 18. Food and Agriculture Organization, Rome, Italy.

[7] Hafner M, Landgraf P, Ludwig J, Rice A, Ojo T, et al (2008) Identification of microRNAs and other small regulatory RNAs using cDNA library sequencing. Methods 44: 3–12. 18158127 10.1016/j.ymeth.2007.09.009 PMC2847350

[8] Burge SW, Daub J, Eberhardt R, Tate J, Barquist L, et al (2013) Rfam 11.0: 10 years of RNA families. Nucleic Acids Research 41: D226–232. 23125362 10.1093/nar/gks1005 PMC3531072

[9] Denman RB (1993) Using RNAFOLD to predict the activity of small catalytic RNAs. Biotechniques 15: 1090–1095. 8292343

[10] Mortazavi A, Williams BA, McCue K, Schaeffer L, Wold B (2008) Mapping and quantifying mammalian transcriptomes by RNA-Seq. Nature Methods 5: 621–628. 18516045 10.1038/nmeth.1226 PMC13303166

[11] Dai X, Zhao PX (2011) psRNATarget: a plant small RNA target analysis server. Nucleic Acids Research 39: W155–159. 21622958 10.1093/nar/gkr319 PMC3125753

[12] Zhao HS, Chen DL, Peng ZH, Wang LL, Gao ZM (2013) Identification and characterization of microRNAs in the leaf of ma bamboo (*Dendrocalamus latiflorus*) by deep sequencing. PLoS One 8: e78755. 24205306 10.1371/journal.pone.0078755 PMC3804618

[13] Bauer S, Grossmann S, Vingron M, Robinson PN (2008) Ontologizer 2.0—a multifunctional tool for GO term enrichment analysis and data exploration. Bioinformatics 24: 1650–1651. 18511468 10.1093/bioinformatics/btn250

[14] Chen C, Ridzon DA, Broomer AJ, Zhou Z, Lee DH, et al (2005) Real-time quantification of microRNAs by stem-loop RT-PCR. Nucleic Acids Research 33 (20) e179. 16314309 10.1093/nar/gni178 PMC1292995

[15] Ding Y, Chen Z, Zhu C (2011) Microarray-based analysis of cadmium-responsive microRNAs in rice (*Oryza sativa*). Journal of experimental botany 62 (10) 3563–3573. 21362738 10.1093/jxb/err046 PMC3130178

[16] Unver T, Budak H (2009) Conserved microRNAs and their targets in model grass species Brachypodium distachyon. Planta 230 (4) 659–669. 19585143 10.1007/s00425-009-0974-7

[17] Livak KJ, Schmittgen TD (2001) Schmittgen, Analysis of relative gene expression data using real-time quantitative PCR and the 2(−Delta Delta C(T)) Method. Methods 25 (4) 402–408. 11846609 10.1006/meth.2001.1262

[18] Pantaleo V, Szittya G, Moxon S, Miozzi L, Moulton V, et al (2010) Identification of grapevine microRNAs and their targets using high-throughput sequencing and degradome analysis. The Plant Journal 62: 960–976. 20230504 10.1111/j.0960-7412.2010.04208.x

[19] Yi R, Zhu Z, Hu J, Qian Q, Dai J, et al (2013) Identification and expression analysis of microRNAs at the grain filling stage in rice (*Oryza sativa* L.)via deep sequencing. PLoS One 8: e57863. 23469249 10.1371/journal.pone.0057863 PMC3585941

[20] Lv S, Nie X, Wang L, Du X, Biradar SS, et al (2012) Identification and characterization of microRNAs from barley (*Hordeum vulgare* L.) by high-throughput sequencing. International journal of molecular sciences 13: 2973–2984. 22489137 10.3390/ijms13032973 PMC3317698

[21] Li YF, Zheng Y, Jagadeeswaran G, Sunkar R (2013) Characterization of small RNAs and their target genes in wheat seedlings using sequencing-based approaches. Plant Science 203–204: 17–24. 10.1016/j.plantsci.2012.12.014 23415324

[22] Song QX, Liu YF, Hu XY, Zhang WK, Ma B, et al (2011) Identification of miRNAs and their target genes in developing soybean seeds by deep sequencing. BMC Plant Biology 11: 5. 21219599 10.1186/1471-2229-11-5 PMC3023735

[23] Bushati N, Cohen SM (2007) microRNA functions. Annual Review of Cell and Developmental Biology 23: 175–205. 10.1146/annurev.cellbio.23.090506.123406 17506695

[24] Carrington JC, Ambros V (2003) Role of microRNAs in plant and animal development. Science 301 (5631) 336–338. 12869753 10.1126/science.1085242

[25] Cuperus JT, Fahlgren N, Carrington JC (2011) Evolution and functional diversification of MIRNA genes. Plant Cell 23: 431–442. 21317375 10.1105/tpc.110.082784 PMC3077775

[26] Zhang B, Pan X, Cannon CH, Cobb GP, Anderson TA (2006) Conservation and divergence of plant microRNA genes. The Plant Journal 46: 243–259. 16623887 10.1111/j.1365-313X.2006.02697.x

[27] Tang G (2010) Plant microRNAs: an insight into their gene structures and evolution. Seminars in Cell and Developmental Biology 21: 782–789. 20691276 10.1016/j.semcdb.2010.07.009

[28] Ma Z, Coruh C, Axtell MJ (2010) *Arabidopsis lyrata* small RNAs: transient MIRNA and small interfering RNA loci within the Arabidopsis genus. The Plant Cell Online 22: 1090–1103. 10.1105/tpc.110.073882 PMC2879747 20407023

[29] Allen E, Xie Z, Gustafson AM, Sung GH, Spatafora JW, et al (2004) Evolution of microRNA genes by inverted duplication of target gene sequences in Arabidopsis thaliana. Nature Genetics 36: 1282–1290. 15565108 10.1038/ng1478

[30] Maher C, Stein L, Ware D (2006) Evolution of Arabidopsis microRNA families through duplication events. Genome research 16: 510–519. 16520461 10.1101/gr.4680506 PMC1457037

[31] Guddeti S, Zhang DC, Li AL, Leseberg CH, Kang H, et al (2005) Molecular evolution of the rice miR395 gene family. Cell Research 15: 631–638. 16117853 10.1038/sj.cr.7290333

[32] Peng ZH, Lu Y, Li LB, Feng Q, Gao ZM, et al (2013) The draft genome of the fast-growing non-timber forest species moso bamboo (*Phyllostachys heterocycla*). Nature Genet 45: 456–461. 23435089 10.1038/ng.2569

[33] Chen Ruiyang (2003) Chromsome atlas of various bamboo species, Chromsome atlas of major economic plants genome in china (Tomus IV). Science Press, Beijing.

[34] He CY, Cui K, Zhang JG, Duan AG, Zeng YF (2013) Next-generation sequencing-based mRNA and microRNA expression profiling analysis revealed pathways involved in the rapid growth of developing culms in Moso bamboo. BMC Plant Biology 13: 119. 23964682 10.1186/1471-2229-13-119 PMC3765735

[35] Wei Q, Liang YH, Li GL (2013) Evolution of miRNA in plants. HEREDITAS (Beijing) 35: 315–323. 10.3724/sp.j.1005.2013.00315 23575537

[36] Unte US, Sorensen AM, Pesaresi P, Gandikota M, Leister D, et al (2003) *SPL8*, an SBP-box gene that affects pollen sac development in Arabidopsis. Plant Cell 15: 1009–1019. 12671094 10.1105/tpc.010678 PMC152345

[37] Zhang Y, Schwarz S, Saedler H, Huijser P (2007) *SPL8*, a local regulator in a subset of gibberellin-mediated developmental processes in Arabidopsis. Plant molecular biology 63 (3) 429–439. 17093870 10.1007/s11103-006-9099-6

[38] Hou H, Li J, Gao M, Singer SD, Wang H, et al (2013) Genomic organization, phylogenetic comparison and differential expression of the SBP-box family genes in grape. PloS One 2013, 8 (3) e59358. 10.1371/journal.pone.0059358 PMC3601960 23527172

[39] Cardon G, Hohmann S, Klein J, Nettesheim K, Saedler H, et al (1999) Molecular characterisation of the Arabidopsis SBP-box genes. Gene 237: 91–104. 10524240 10.1016/s0378-1119(99)00308-x

[40] Yang Z, Wang X, Gu S, Hu Z, Xu H, et al (2008) Comparative study of SBP-box gene family in Arabidopsis and rice. Gene 407: 1–11. 17629421 10.1016/j.gene.2007.02.034

[41] Ashburner M, Ball CA, Blake JA, Botstein D, Butler H, et al (2000) Gene ontology: tool for the unification of biology. The Gene Ontology Consortium. Nature Genetics 25: 25–29. 10802651 10.1038/75556 PMC3037419

[42] Liu M, Qiao G, Jiang J, Yang H, Xie L, et al (2012) Transcriptome sequencing and de novo analysis for ma bamboo (*Dendrocalamus latiflorus Munro*) using the Illumina platform. PloS one 7 (10) e46766. 23056442 10.1371/journal.pone.0046766 PMC3463524

[43] Gao ZM, Li CL, Peng ZH (2011) Generation and analysis of expressed sequence tags from a normalized cDNA library of young leaf from Ma bamboo (*Dendrocalamus latiflorus Munro*). Plant cell reports 30 (11) 2045–2057. 21713530 10.1007/s00299-011-1112-0

[44] Zhao HS, Peng ZH, Fei BH, Li LB, Hu T, et al. (2014) BambooGDB: a bamboo genome database with functional annotation and an analysis platform. Database bau006. 10.1093/database/bau006 PMC3944406 24602877

