# Supplementary material for: Discovery and Comparative Profiling of microRNAs in Representative Monopodial Bamboo (Phyllostachys edulis) and Sympodial Bamboo (Dendrocalamus latiflorus)
Source: PLoS One. 2014 Jul 11;9(7):e102375. doi: 10.1371/journal.pone.0102375 (PMC4094515; doi:10.1371/journal.pone.0102375)
Supplement: File S1 — Primers for qRT-PCR and PCR of miRNAs. (DOC) [file pone.0102375.s001.doc]

Additional file 1. Primers for qRT-PCR and PCR of miRNAs

| miRNA | Primer | Sequence (5' to 3') |
| --- | --- | --- |
| miR396 | RT Primer | CTCAACTGGTGTCGTGGAGTCGGCAATTCAGTTGAGCAGTTCAA |
|  | Forward Primer | ACAGGCGATCCACAGGCTTTC |
| miR397 | RT Primer | CTCAACTGGTGTCGTGGAGTCGGCAATTCAGTTGAGCATCAACG |
|  | Forward Primer | ACGGGCGATCATTGAGTGCAG |
| miR1432 | RT Primer | CTCAACTGGTGTCGTGGAGTCGGCAATTCAGTTGAGTGTCGGTG |
|  | Forward Primer | ACGGGCGATTCAGGAGAGATG |
| miR7748 | RT Primer | CTCAACTGGTGTCGTGGAGTCGGCAATTCAGTTGAGACCGTCTG |
|  | Forward Primer | ACGGGCGAGATATGTTGGCCA |
| Universal Reversal Primer | | CTCAACTGGTGTCGTGGAGTC |
| U6 | Forward primer | GGACATCCGATAAAATTGGAACGATACAG |
|  | Reverse Primer | AATTTGGACCATTTCTCGATTTATGCGTGT |
