# Supplementary material for: Discovery and Comparative Profiling of microRNAs in Representative Monopodial Bamboo (Phyllostachys edulis) and Sympodial Bamboo (Dendrocalamus latiflorus)
Source: PLoS One. 2014 Jul 11;9(7):e102375. doi: 10.1371/journal.pone.0102375 (PMC4094515; doi:10.1371/journal.pone.0102375)
Supplement: File S2 — Primers for the precursor amplification of miRNAs. (DOC) [file pone.0102375.s002.doc]

Addition file 2. Primers for precursor amplification of miRNAs

| miRNA | Primer | Sequence (5' to 3') |
| --- | --- | --- |
| miR396 | pre-396-F | CATCTTCTCTCTCTCTTTCTATC |
|  | pre-396-R | GAACTCCCTAAAAACACAGAATTG |
| miR397 | pre-397-F | ACTCACTCACTCCTAGCTACCTG |
|  | pre-397-R | ATGCTAGCACTAGGCTCCGTAC |
| miR1432 | pre-1432(1)-F | GCCTATATATAGACACAGTGTGTG |
|  | pre-1432(1)-R | CACCTGCAAGATCAACATAGAG |
| miR7748 | pre-7748-F | TAATCCCTCATCACACATGGTTTC |
|  | pre-7748-R | TATGTGCACCAACCGTTTGTGATAT |
