# Supplementary figures and images for: Discovery and Comparative Profiling of microRNAs in Representative Monopodial Bamboo (Phyllostachys edulis) and Sympodial Bamboo (Dendrocalamus latiflorus)
Source: PLoS One. 2014 Jul 11;9(7):e102375. doi: 10.1371/journal.pone.0102375 (PMC4094515; doi:10.1371/journal.pone.0102375)

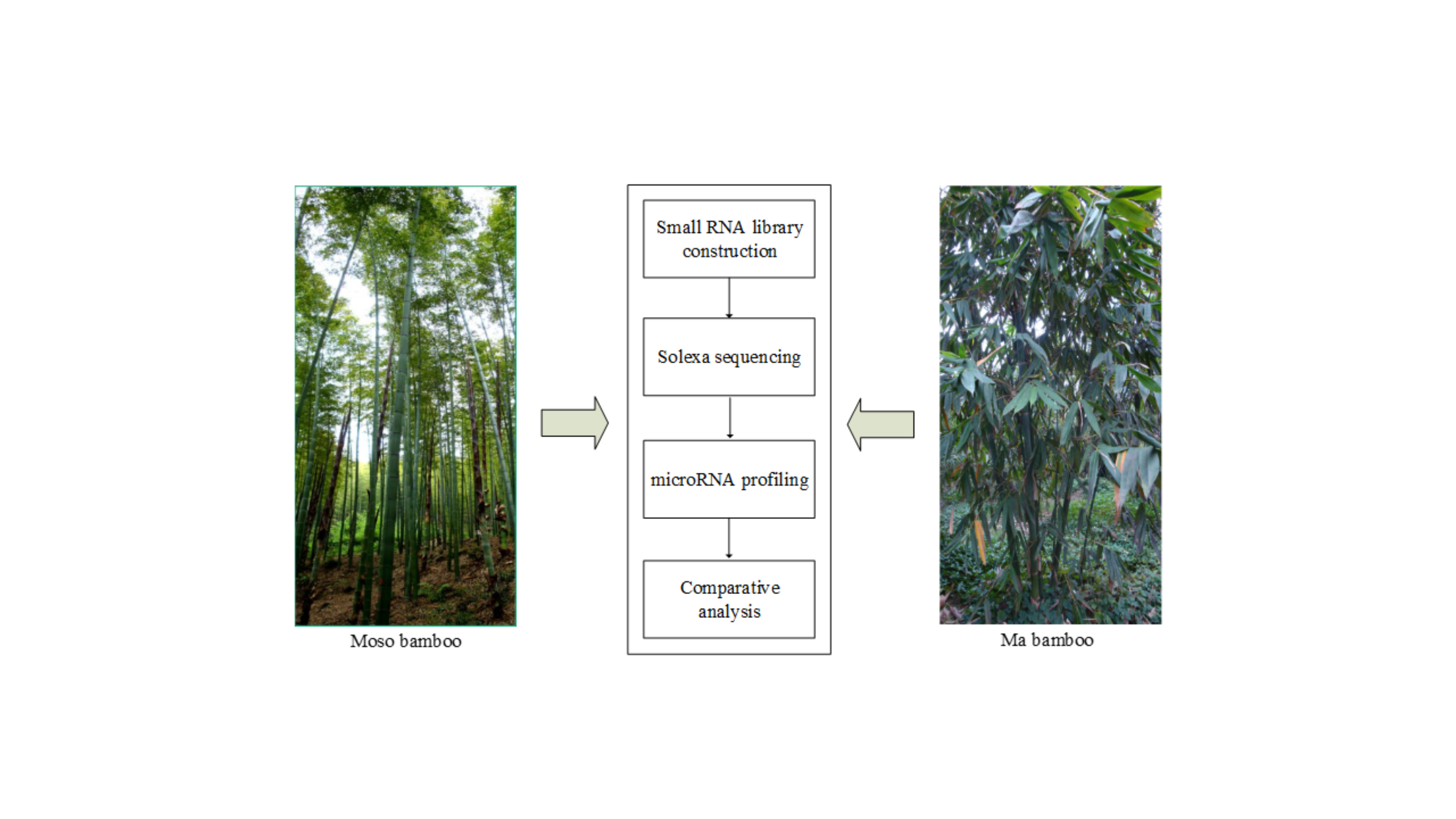

Supplement: File S3 — Dramatically different phenotype between moso bamboo and ma bamboo. (TIF) [file pone.0102375.s003.tif]

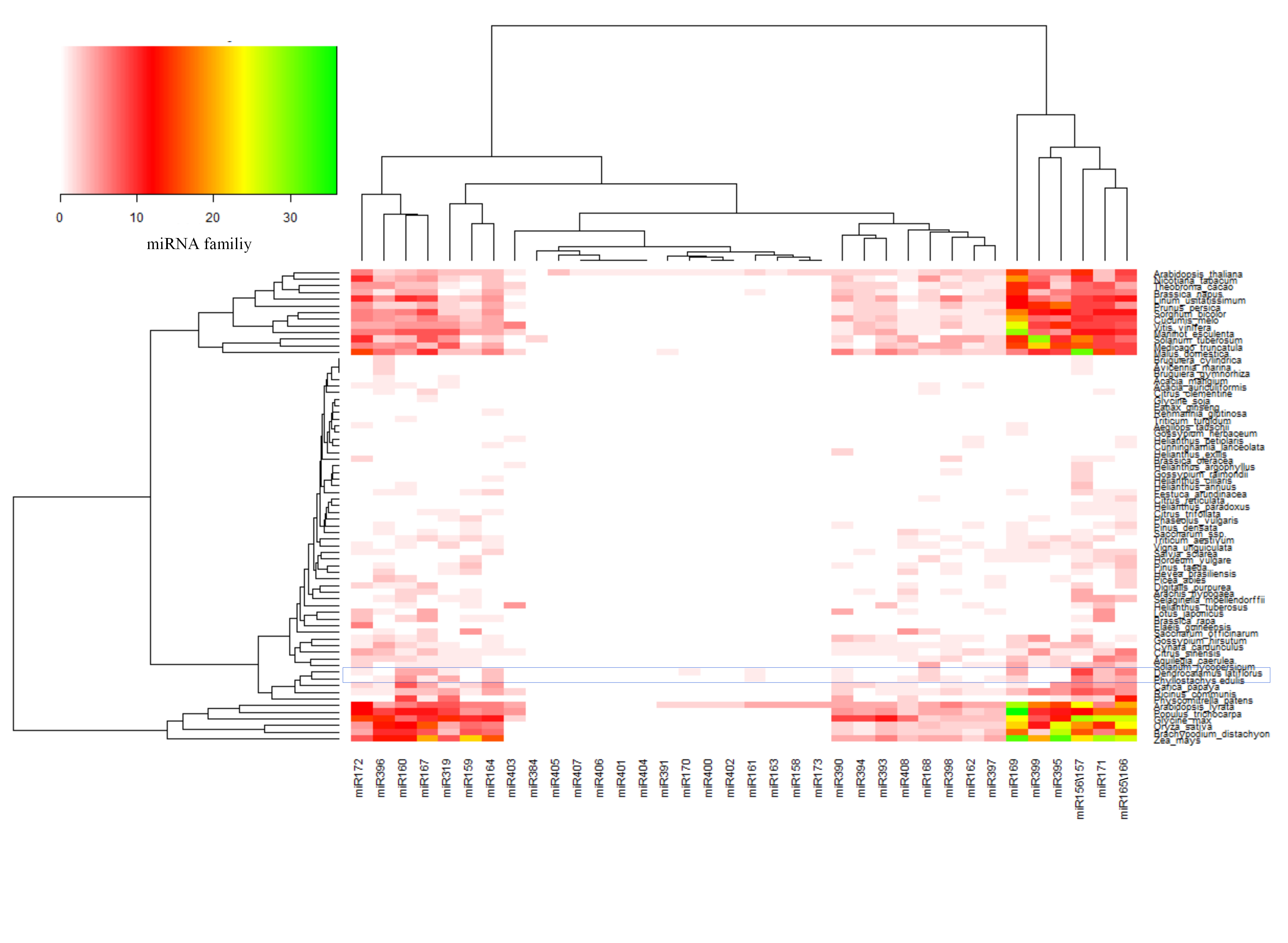

Supplement: File S5 — Heat map of highly conserved miRNA family in plants. (TIF) [file pone.0102375.s005.tif]
