# Supplementary material for: Discovery and Comparative Profiling of microRNAs in Representative Monopodial Bamboo (Phyllostachys edulis) and Sympodial Bamboo (Dendrocalamus latiflorus)
Source: PLoS One. 2014 Jul 11;9(7):e102375. doi: 10.1371/journal.pone.0102375 (PMC4094515; doi:10.1371/journal.pone.0102375)
Supplement: File S8 — Expression analysis of miRNAs in leaves of ma bamboo using qRT-PCR. (DOC) [file pone.0102375.s008.doc]

# Error bars representing the standard deviation were derived from the three experiments in triplicate.
