# Supplementary material for: Discovery and Comparative Profiling of microRNAs in Representative Monopodial Bamboo (Phyllostachys edulis) and Sympodial Bamboo (Dendrocalamus latiflorus)
Source: PLoS One. 2014 Jul 11;9(7):e102375. doi: 10.1371/journal.pone.0102375 (PMC4094515; doi:10.1371/journal.pone.0102375)
Supplement: File S9 — Sequencing results of mature miRNAs. (DOC) [file pone.0102375.s009.doc]

Addition file 9. Sequencing results of mature miRNAs

| miRNA | **Sample** | **Sequence** (5' to 3') |
| --- | --- | --- |
| miR396 | **Moso bamboo** | **acaggcgatccacaggctttcTTGAACTGctcaactgaattgccgactccacgacaccagttgag** |
|  | **Ma bamboo** | **acaggcgatccacaggctttcTTGAACTGctcaactgaattgccgactccacgacaccagttgag** |
| miR397 | **Moso bamboo** | **acgggcgatcattgagtgcagCGTTGATGctcaactgaattgccgactccacgacaccagttgag** |
|  | **Ma bamboo** | **acgggcgatcattgagtgcagCGTACGTATctcaactgaattgccgactccacgacaccagttgag** |
| miR1432 | **Moso bamboo** | **acgggcgattcaggagagatgACACCGACActcaactgaattgccgactccacgacaccagttgag** |
|  | **Ma bamboo** | **acgggcgattcaggagagatgGAGGCGAGGctcaactgaattgccgactccacgacaccagttgag** |
| miR7748 | **Moso bamboo** | **acgggcgagatatgttggccaCCACAGACGGTctcaactgaattgccgactccacgacaccagttgag** |

Note: Mature miRNAs were in red letters, the mismatched bases were marked in yellow.
