# Supplementary material for: Discovery and Comparative Profiling of microRNAs in Representative Monopodial Bamboo (Phyllostachys edulis) and Sympodial Bamboo (Dendrocalamus latiflorus)
Source: PLoS One. 2014 Jul 11;9(7):e102375. doi: 10.1371/journal.pone.0102375 (PMC4094515; doi:10.1371/journal.pone.0102375)
Supplement: File S10 — Sequencing result of sequence containing miRNA precursor in moso bamboo. (DOC) [file pone.0102375.s010.doc]

Addition file 10. Sequencing result of sequence containing miRNA precursor in moso bamboo

| miRNA | **Sequence** (5'to3') |
| --- | --- |
| miR396 | catcttctctctctctttctatctctagtctctacacgtctttctctttttctctctctatctcttgctctttcggttgttgctgctgtgtgccttgatgtgatgattcaggcaggcgctcgcgagaattgtggtgtggtgtgttccttccgtccgatggcaaagaaagatgcgcgggcatgctttccacaggctttcttgaactgtgaactcgtggggtggggatttgtagaatcaagaatatggtggatggcttcgtgtgagttcaattatctctgaattgtggagctctcggtttctctcttgtcatctctacctgttgatggttcaagaaagcccatggaaaccatgcagcgtcttgcgtttcgttcattccgttccgcggagaattcttgctaacaattctgtgtttttagggagttc |
| miR397 | actcactcactcctagctacctgtagactgtagtacaaatggggctacctgtctcaaaagctaatcgaagattcctgcgtttatgtctgttcggaagcacaaattcggtgtggctgagaaatcaaatggaagaggaagagaagcaaaggcatcattgagtgcagcgttgatgaaccggccggcgagcggccgtgcatccacggtcaatcttccgctcaccagcgctgcactcaatcacgcctttgctctctctggcggtgcagccgtgttggtactcatcagtcgtcaggttttgttccatctggcctttttcttccctgatcgatctgtcttacaggctatccggctgcaggcagcagtactagctagttcgttaatccacttgttagtgtacggagcctagtgctagcat |
| miR1432 | gcctatatatagacacagtgtgtgtgcgcattatgcaggaacacacacaaaaatagcttagtgtatatgcagagagccagagagcaagaagcacatagtagcagatatgcagttcatacaagagatcagcagaagagggcgtactgttcttgtggggtttgggacctgtgttcaggagagatgacaccgacatcaaatggattgatttggactatgtccacccgtttgaagtgggtgtcatctcgcctgaacataggaatcactatccctgaataatgaatgcatacctgatacctcgacgggtcttcttctagttctacacactttcttgttcttgatccgcgttggcgagtgctttacaagaatacacaaggtaatgcaacagcctcttcagacttcacttgtcctttcgttcttctctatgttgatcttgcaggtg |
| miR7748 | taatccctcatcacacatggtttctgaaccgtctgatttattgtgtatataataacaggagattatcacatgcggtttttgagccgtctgtgatagttcacaacacatatggtacgtataaataaccatatgtgaacataactatcatagacggcttcgaccacaaaacagtctgtgatatgtcggccatcacagacggttgtgtttaccaacgatctgtgatatgttggccaccacagacggttgtgtttacaaaccatctgtgactgactacatatcatagacggttggtgcatagaaccgtctgtgatagtaggcgttcatagacggtttataccacataaccgtctgtgatattcgttcatcacagatggttgtgtttacaaaccgtctgtgataggctacatatcacaaacggttggtgcacata |

Note: miRNA precursors were in red letters.
